# New Horizons in Immunology and Immunotherapy of Acute Leukemias and Related Disorders

**DOI:** 10.3390/cancers15092422

**Published:** 2023-04-23

**Authors:** Francesco Lanza, Michela Rondoni, Beatrice Anna Zannetti

**Affiliations:** Hematology Unit and Metropolitan Romagna Transplant Network, University of Bologna, 40126 Ravenna, Italy; michela.rondoni@auslromagna.it (M.R.); beatriceanna.zannetti@auslromagna.it (B.A.Z.)

Accumulating data have shown that molecular aberrations have the potential to trigger the development of acute leukemia, and that the routine application of novel molecular biology technologies has facilitated the development of investigational drugs which target driver genetic mutations. In the last decade, a great number of clinical trials have been testing “druggable” mutations in the context of novel targeted therapies [1,2].

More recently, the importance of tumor (including bone marrow) microenvironment and immunologic host effectors have been subjected to an increasing number of clinical and biological studies. These have aimed to identify the interplay between acute leukemia blasts, the hematopoietic niche, and the cells of the immune system. A better assessment of these systems has contributed to the clarification of the pathogenesis of acute myeloid leukemia (AML) development and growth as well as to the mechanisms underlying the ability of leukemic cells to promote immunological escape and systemic tolerance. It has become evident that tolerogenic pathways may be responsible for an immunosuppressive microenvironment, one which is capable of negatively influencing anti-leukemia immune responses and of inducing resistance to chemotherapy [3,4].

This Special Issue of *Cancers* (entitled “Cancer Immunology and Immunotherapy”) is focused on both the biological and clinical applications of immunotherapy in a variety of tumors, including hematological malignancies. This editorial will attempt to highlight key issues and crucial developments in the areas of AML, acute lymphoblastic leukemia (ALL), and related disorders, drawing from manuscripts published in the years 2022 and 2023 in *Cancers* to do so.

Interestingly, some of these metabolic pathways can be targeted by new drugs, such as immune checkpoint and macrophage checkpoint inhibitors [3,4]. This is a novel therapeutic tool thought to be associated with increased anti-tumor immunity. However, it must be said that a great number of immune and therapeutic interventions may be used in acute leukemia, including monoclonal antibodies (such as CD33, CD123, TIM3, CD244, CLL1, etc.), T-cell engagers (bispecific antibodies targeting CD33 and CD123, including either BiTesor BiKes, and dual-affinity retargeting antibodies), tandem diabodies, adoptive T-cell therapies, CAR-T cells (CD123 CAR-T, CD33 CAR-T, FLT-3 CAR-T, CLL1 CAR-T, CD44v6 CAR-T), adoptive NK-cell therapies (haploidentical NK cells, NK-cell activation, ex vivo NK-cell expansion), checkpoint blockade and macrophage checkpoint blockade, and leukemia vaccines [3,4,5].

Recent data seem to demonstrate that the involvement of a great number of immunotherapy strategies in implementing patients’ immunity against blast cells may be of clinical utility; in particular, the role played by anti-leukemia vaccines to control acute leukemia progression has been documented in several studies [6,7]. Data collected thus far in the field of vaccine-based trials seem to indicate the efficacy of vaccinating AML. Furthermore, the clinical use of immune checkpoints and macrophage checkpoint blockades may enhance our understanding regarding the value of anti-leukemia vaccines in clinical practice. For example, Wilms’ tumor 1 (WT1) has been increasingly used in experimental studies for AML [3,6]. WT1 is markedly overexpressed in more than 90% of AML cases [8], being an attractive antigen for use as a target for vaccination purposes in AML. The identification of immunogenic WT1 epitopes, capable of eliciting anti-leukemia cytotoxic CD8^+^ T cells, was recently reported in a few studies, thus providing the rationale for the development of clinical studies using WT1 vaccines. In some preliminary studies, a WT1 peptide vaccine was capable of inducing WT1-specific cytotoxic CD8^+^ T cells, which resulted in disease improvement [5,6]. Interestingly, no severe side effects were observed during the performance of vaccination protocols in adult and elderly patients. Based on data collection from phase I studies in AML, the prolonged WT1 peptide vaccination seems to be associated with sustained MRD negativity. In our view, two clinical settings are worthy of being investigated in AML for vaccination strategies: the post-induction consolidation phase and post-allogeneic stem cell transplantation (SCT) phase, especially in those patients showing persistent MRD positivity. Both situations represent the ideal setting to better assess the therapeutic efficacy of vaccines for the prevention of relapse as well as for a formal demonstration of this approach as an immunological maintenance strategy. New vaccination strategies have been developing in more recent years based on results obtained from the use of peptide and dendritic cells. These have been associated with better outcomes as compared to the historical control, especially in the AML subset characterized by the capacity to activate a marked and sustained immune response to WT1. Furthermore, the patients who achieved a better response rate to vaccination showed an increased frequency of WT1-specific cytotoxic T-lymphocytes (CTLs) before vaccination. In a further study, a T-cell receptor gene therapy targeting WT1 was able to prevent AML relapse post-allogeneic SCT [6,7,9,10,11].

In the manuscript of Riillo et al. [12], the use of a novel bispecific T-cell engager (CD1a-CD3ε) BTCE was tested in cortical-derived T-cell acute lymphoblastic leukemia (T-ALL) blasts. It is well known that T-ALL is an aggressive variant of acute leukemia characterized by a poor response to standard chemotherapy, a high incidence of relapse and an unfavorable prognosis. In contrast to B-ALL, where the availability of several immunotherapeutic strategies such as blinatumomab and inotuzumab [13] has dramatically improved the outcomes of the treatment of these leukemia categories, the lack of tumor-restricted T-cell antigens still hampers treatment progress in T-ALL. In this paper, a novel asymmetric (2 + 1) bispecific T-cell engager (BTCE), targeting CD1a and CD3ε (CD1a-CD3ε) starting from the development of a novel mAb named UMG2, was developed and tested in the clinics. UMG2 mAb reacted against CD1a, a glycoprotein highly expressed by cortical T-ALL cells. CD1a-CD3ε showed the capacity to induce in vitro high-T-cell-mediated cytotoxicity against blasts taken from CD1a + T-ALL. Moreover, in a PBMC-reconstituted NGS mouse model bearing human T-ALL, CD1a-CD3ε significantly inhibited the growth of human T-ALL xenografts, translating in a significant survival advantage for treated animals. Based on these findings, the authors documented that CD1a-CD3ε may represent a novel BTCE highly active against CD1a-expressing cortical-derived T-ALL cells.

Interestingly, as an alternative to checkpoint inhibitors (ICIs) constituted by monoclonal antibodies (mAbs), the concept of active immunization with vaccines has been reported in two manuscripts obtained from this series from Cancers.

In the first paper included, published by Yusuke Oji [14], a promising novel immunotherapeutic tool was proposed. It was based on a WT1 trio peptide-based cancer vaccine for rare cancers expressing the shared target WT1. WT1 is expressed in rare cancers, as well as in several acute myeloid leukemic cells, which may overexpress this molecule. The results showed that the biweekly WT1 trio peptide vaccine, comprising two WT1-cytotoxic T lymphocyte (CTL) peptides and one WT1-helper T lymphocyte peptide, induced stronger immune responses targeting WT1 than the weekly WT1-CTL peptide (WT1-235) vaccine. The safety profile of this agent was acceptable, except for in the case of the grade 3 myasthenia gravis-like symptoms observed in a single patient. A total of 15 (33.3%) of the 45 patients with recurrent or advanced rare cancers, including malignant glioma, soft-tissue sarcoma, and malignant pleural mesothelioma, achieved stable disease status after 3 months of treatment. Based on these preliminary data, it can be postulated that in cancer types overexpressing WT1, WT1-targeted immunotherapy may be effective in reducing tumor burden and achieving a remission state for the disease.

Interestingly, as an alternative to checkpoint inhibitors mAbs, the concept of active immunization with vaccines was further discussed by Tobias [15], who reported combined vaccination with B-cell peptides targeting Her-2/neu and immune checkpoints as a treatment strategy in several cancers. Recently, it has been shown that mimotopes, representing the B-cell epitope of therapeutic mAbs, proved capable of inducing immunological memory and producing antibodies with similar functionality to the respective mAbs/ICIs. It is well known that tumor therapies with mAbs targeting tumor-associated antigens (TAAs) or immune checkpoint inhibitors (ICIs) have revolutionized treatment for several oncological and hematological malignancies. However, the development of resistance and/or response failure may occur in several circumstances, limiting the clinical efficacy of ICIs. Based on these considerations, the use of active immunization with vaccines may represent a therapeutic option. The paper presently under consideration discussed recent clinical trials involving combination therapies with mAbs targeting the PD-1/PD-L1 axis and Her-2/neu, treatments which were used as paradigm for the clinical employment of TAA. A new frontier of cancer treatment via active immunization based on the use of combined mimotopes/B-cell peptides targeting a variety of immunological molecules may open new concept in cancer biology and therapy.

Another area of investigation attracting a number of clinical studies across the world is represented by the assessment of immune-related adverse events during ICIs administration or during vaccine-based therapeutic interventions.

In the paper by Garrison et al. [16], the authors investigated a novel potential role for monocytes. This was revealed by single-cell analysis of immunotherapy-induced immune-related side effects. It is well established that the use of immunotherapy is associated with a significant risk of a variety of immune-based side effects. These adverse events are well known, but remain poorly understood from a pathogenetic point of view. To better understand the main cellular players involved in the development of side effects during ICIs, the authors have analyzed single-cell sequencing data from PBMCs drawn from patients who developed skin immune related side effects. Based on these results, it was shown that monocytes play a pivotal role in the development of ICI-related side effects. This study is an important step in improving our understanding of the mechanisms that drive immune related adverse events.

In a review article by Shalit et al. [17], entitled “Predictive Biomarkers for Immune-Related Endocrinopathies following Immune Checkpoint Inhibitors Treatment”, there was a discussion of the different predictive biomarkers, such as cytokines, human leukocyte antigens (cluster of differentiation), mAbs, and hormones, and blood cells such as eosinophils that could potentially be used in clinical practice in order to predict immune-related adverse effects and manage them appropriately. It is well known that endocrine dysfunctions affect around 10% of all treated patients and that they can be successfully treated if discovered early but that they can be life-threatening if diagnosed late. Thus, it is crucial to identify predictive biomarkers that might indicate the risk of development of endocrinopathies and can guide clinical decisions. Endocrine dysfunctions can manifest as thyroid dysfunction, hypoparathyroidism, primary adrenal insufficiency, hypophysitis, and insulin-deficient diabetes mellitus. Currently, there are several clinical trials investigating predictive biomarkers for immune-related adverse effects. The design of most clinical trials is based on the collection of a variety of biological specimens (tissue biopsy, blood, plasma, saliva, and stool) at baseline and regular intervals during treatment [17].

In summary, this Special Issue of *Cancers* includes a collection of articles covering topics in basic, translational and clinical studies into several types of cancers, including hematologic malignancies, that will be of interest for a wide audience of researchers and clinicians working on this field. It is now well established that, despite the variety of mechanisms used by immunotherapies, these treatments require fidelity and accuracy in the identification of antigens in order to direct cell killing and avoid the occurrence of off-target effects. The best application of immunotherapy in AML may be represented by its use in combination with chemotherapy as a bridge to allogenic SCT or for those patients who are unfit or frail and therefore are not candidates to receive standard induction chemotherapy.

It is probable that immunotherapy will became more and more important in the treatment scenario of acute leukemia in the coming years. Indeed, it may contribute to the implementation of precision medicine and to improving the clinical management of acute leukemia.
